# Radix Entomolaris and Complex Incisor Anatomy in a Saudi Cohort: A Retrospective Study

**DOI:** 10.3390/diagnostics15131721

**Published:** 2025-07-06

**Authors:** Mubashir Baig Mirza

**Affiliations:** Conservative Dental Science Department, College of Dentistry, Prince Sattam Bin Abdulaziz University, Al-Kharj 11942, Saudi Arabia; m.mirza@psau.edu.sa; Tel.: +966-593492190

**Keywords:** incisors, mandible, molars, root canal morphology, Saudi Arabia

## Abstract

**Background/Objectives:** A thorough understanding of tooth anatomy is essential for effective root canal treatment. This study aims to investigate the root canal morphology of mandibular incisors (MIs) and the presence of distolingual roots in mandibular first molars (MFMs) and to explore the potential correlation between these anatomical variations. **Methods:** A total of 562 CBCT scans were retrospectively analyzed, corresponding to 1124 mandibular central incisors (MCIs), mandibular lateral incisors (MLIs), and MFMs each. The DLR in MFMs was correlated with the complex anatomy in MIs and analyzed using a chi-square test, with the odds ratio obtained through binary regression analysis. Differences related to gender, site, and age were analyzed using the chi-square test. **Results:** Most MI scans revealed Vertucci Type I canal morphology, with a higher percentage in MCIs (71.1%) than MLIs (64.9%). Additionally, 5.25% of MFM scans indicated a DLR, with a higher prevalence in males (3.5%) and younger individuals (3.4%); however, a statistically significant difference was observed only in MCIs (*p* = 0.035) across different age groups. The study also identified a highly significant difference in complex canal anatomy, comparing both MIs on either side and the presence of DLR in MFMs (*p* < 0.001). Furthermore, the relationship between complex canal systems in MIs and MFMs with DLR was confirmed. **Conclusions:** In conclusion, the Vertucci Type I canal configuration was predominant in both MIs, followed by Type III. The DLR was present in 5.25% of the total scans, and its presence strongly correlated with complex morphology in both MIs.

## 1. Introduction

The literature extensively documents the intricate nature of root canal morphology among various teeth. It is imperative to comprehend these intricacies, as overlooked canals may serve as a habitat for numerous microorganisms, potentially resulting in treatment failure [1]. The previously assumed simple morphologies of the teeth have now been acknowledged as more complex [2]. The advancement of CBCT as a diagnostic tool in endodontics has played a crucial role in enhancing our understanding of these complexities within the oral and maxillofacial region. Its three-dimensional (3D) view significantly impacts our ability to recognize and navigate these complexities [3]. Additionally, lower radiation, following the as low as diagnostically acceptable (ALADA) protocol, combined with its non-invasiveness and the ability to visualize internal anatomy in three different planes—coronal, sagittal, and axial has made it a popular choice among root canal specialists [4]. However, it should be regarded as a supplementary tool in endodontic practice and used only when absolutely necessary [5].

Root canals are primarily categorized using the Vertucci system, which is widely utilized within the realms of research, academia, and endodontic practice. Nevertheless, newer classifications, such as the Ahmed et al. system, have garnered recognition due to their careful consideration of internal anatomies [6]. This newer system is more inclusive, considering not only the number of canals but the number of roots and canal configurations for each root as well [7]. Such a classification is easier to understand without having to memorize a preset classification, as given by Vertucci [8].

Extensive research into the morphology of the mandibular incisors (MIs) has demonstrated diverse internal morphologies, contradicting their seemingly uncomplicated external characteristics [9]. In this regard, a recent worldwide meta-analysis reported the presence of an extra-lingual canal ranging from 21.9% in central incisors to 26% in lateral incisors [10]. Moreover, research among the Turkish and Chinese populations has pointed to an additional root in the mandibular lateral incisors, albeit to a lesser extent [11,12].

The number of roots in teeth gradually increases from the anterior to the posterior region. Mandibular first molars (MFMs), the most common endodontically treated tooth, typically possess two roots, mesial and distal, accommodating three canals [13]. In certain instances, molars may exhibit four canals within their two roots or may even possess an additional distolingual root (DLR) situated towards the distolingual aspect [14]. Molars with DLR are commonly denoted as radix entomolaris (RE), which typically demonstrates a shorter, more conical morphology with more curvature than the distobuccal root, making root canal treatment (RCT) challenging [15]. Clearly, a thorough understanding and astuteness are required to recognize and treat these teeth effectively to prevent the development of reinfection or persistence of symptoms.

Recently, a growing interest is directed towards exploring a potential association between the presence of RE in the MFM and the complex anatomy of the MI. Investigations about the same with varying results have been carried out in Taiwan, Korea, China, and India [16,17,18,19,20]. While the literature has investigated root canal morphology across different teeth within the Saudi population, the potential correlation between canal complexities in MIs and the presence of DLR in MFMs remains unexamined. This study aims to (1) characterize root canal morphology in MIs and RE prevalence in MFMs, and (2) investigate their correlation within a Saudi cohort, accounting for demographic variables. It addresses a significant gap in existing literature regarding these anatomical features.

## 2. Materials and Methods

### 2.1. Ethical Approval

The CBCT scans in the College of Dentistry, Prince Sattam bin Abdulaziz University (PSAU) database were analyzed retrospectively (August 2021 to December 2024) after permission from relevant authorities and ethical approval from the Standing Committee for Bioethics Research (SCBR-225/2024, 1 February 2024).

### 2.2. Sample

The research involved analyzing randomly selected scans that included a complete set of MIs and bilateral MFMs. Additionally, only scans from patients aged 18 to 65 years were included, as individuals under 18 were excluded due to concerns related to root completion, and those over 65 were omitted because of an increased likelihood of calcifications. Scans of teeth exhibiting deep carious lesions, signs of external or internal resorption, as well as those with extensive metallic restorations or subjected to previous root canal therapy, were excluded from the study due to concerns about image resolution. Sample size calculation was performed using 3.1.9.7 version G*Power analysis with an effect size of 0.05 and power of 0.8.

### 2.3. CBCT Unit and Evaluation

The Carestream CS9300 (Carestream Dent LLC, Atlanta, GA, USA) CBCT unit used at PSAU is operated following the ALADA principle with the current flow of 5.6 mA at 90 KV tube voltage. A voxel size of 0.2 mm was used, which offers good diagnostic accuracy for identifying root canal morphology. A smaller 8 × 5 cm field of view (FOV) was used, which provides higher resolution and sharper images. A total of 562 scans that met the inclusion criteria were thoroughly examined and analyzed using the RadiAnt Dicom Viewer 2022.1.1 (Medixant, Poznan, Poland) software by an experienced endodontist proficient in utilizing CBCT for the identification and categorization of root canals. The morphologies of MIs, the presence of DLR in MFMs, and the correlation between them were evaluated. The intra-operator calibration validated the accuracy of the interpretation. A kappa value of 0.935 was achieved for intra-observer agreement. For images where inconsistencies appeared, opinion from another Endodontist and an experienced oral and maxillofacial radiologist was sought, ensuring the highest level of reliability in the findings.

### 2.4. Statistical Analysis

All statistical analyses were performed using SPSS statistical software (Version 22, IBM, Chicago, IL, USA) with a significance level set at *p* < 0.05. Descriptive data for MI were reported as frequencies and percentages and analyzed using the Chi-Square test. Variances concerning age, gender, and site were also evaluated in frequencies and percentages and analyzed using the chi-square test. The relationship between the complexity of root canal configuration in MIs and the presence of DLR in MFMs was examined using the chi-square test and odds ratios obtained from binary logistic regression.

## 3. Results

### 3.1. Demographic Data

**Figure 1:**
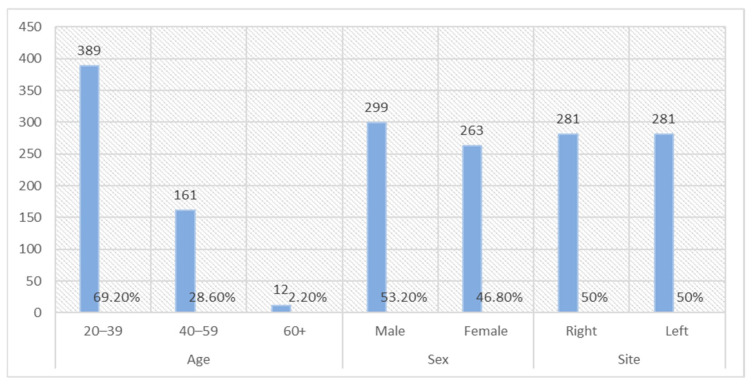
Demographic data.

As seen in Figure 1, 562 scans, including a complete set of MI and MFM scans, were evenly distributed, with 281 on each side.

Among the scans, 53.2% were from male participants, and the remaining 46.8% were from female participants. Most of the scans (69.2%) were from young participants aged 20–39 years, followed by those in the 40–59 age group (28.6%). Only 2.2% of the scans were from participants aged 60 years or older. The average age of the participants was 33.3 years for males and 39.7 years for females.

### 3.2. Root Canal Classification of the MI

Table 1 depicts the evaluation of the MI’s root canal morphology using the Vertucci and Ahmed et al. classification systems.

In most scans, both CI and LI on each side showed a Vertucci Type I canal morphology, indicating a single canal from the orifice to the apex, classified as ^1^TN^1^ by the Ahmed et al. system. The average percentage of Type I morphology in CI (71.15%) was slightly higher than in LI (64.9%). Type III canal morphology, where one canal divides into two along its length and then merges into one before exiting the apex, represented as ^1^TN^1−2−1^ by the Ahmed et al. system, was observed with a mean percentage of 20.8% in LI and 16.2% in CI. Following this, Type V canal morphology was observed, where one canal divides and exits as two at the apex, represented as ^1^TN^1−2^ by the classification system of Ahmed et al. This was observed almost equally in CI (12.65%) and LI (10.85%). Vertucci Type II root canal morphology, represented as ^1^TN^2−1^ by the Ahmed et al. classification system, was only observed in LI (3.45%), where two canals originate at the orifices and merge into one before exiting the root. The differences between the different root canal morphologies were highly significant (*p* < 0.001).

Teeth were further classified as having simple anatomies if they had a single canal (Type I Vertucci) and complex anatomies if they had more than one canal (Types II, III, V) or any variation in the number of roots. As represented in Table 2, the complex anatomies in LI were higher (35.1%) compared to CI (28.9%), and the opposite holds true for simple anatomies, with 71.1% in CI and 64.9% in LI. The differences between them were statistically significant, with *p* = 0.014.

### 3.3. Prevalence of DLR in MFM (Radix)

**Figure 2:**
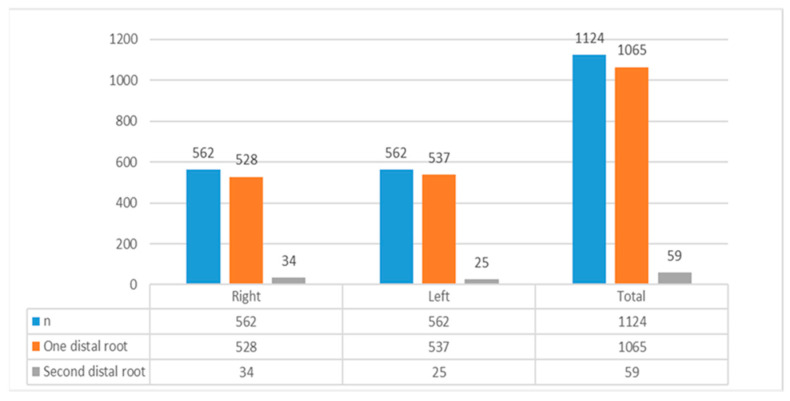
Prevalence of distolingual root in mandibular first molars (MFM). N, total number of MFM; one distal root, presence of only one root on the distal side of the MFM; second distal root, presence of an additional distolingual root (DLR) in the MFM.

Fifty-nine of the total MFM (*n* = 1124) exhibited an additional root distally, accounting for 5.25%. Figure 2 illustrates the distribution of these molars among various genders and age groups, as well as between the right and left quadrants.

**Figure 3:**
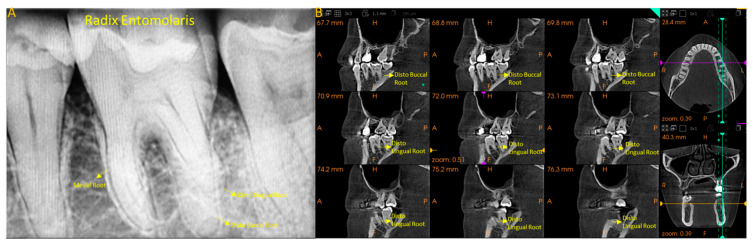
Radix Entomolaris. (**A**), intraoral periapical radiograph of MFM showing three roots: mesial, distobuccal, and distolingual (DLR); (**B**), CBCT image of the same showing the presence of a separate DLR.

Figure 3 presents the MFM with additional DLR represented in periapical and CBCT images.

Table 3 presents a detailed breakdown of Radix entomolaris distribution according to demographic factors, highlighting its comparisons against simple (Type I) and complex (Type III) incisor anatomy.

#### 3.3.1. Gender

Of the 5.25% Radix molars, 3.5% were seen in males, and 1.8% were seen in female patients, as Table 3 shows.

Correlation with CI: 2.7% of the molars with radix in male patients had complex anatomies in CI, compared to 1.2% in female patients. Additionally, 0.8% of male and 0.6% of female patients correlated with simpler anatomy.

Correlation with LI: 2.6% of the molars with RE in male patients correlated with complex anatomies in LI, while 1.3% correlated in female patients. Moreover, 0.9% of male patients and 0.5% of female patients correlated with simpler anatomy. However, comparative analysis using the chi-square test revealed no statistically significant difference between genders in both MCIs (*p* = 0.33) and MLIs (*p* = 0.72), as seen in Table 3.

#### 3.3.2. Age

Table 3 shows that 3.4% of molars with DLR were found in young individuals, 1.5% in middle-aged individuals, and 0.4% in the elderly age group.

Correlation with CI: In young individuals, 2.8% of RE correlated with complex anatomies in the CI, while 1% showed the same in middle-aged individuals. Conversely, in the elderly, higher percentages of radix correlated with simpler anatomies in CI. A statistically significant difference (*p* = 0.035) was observed when comparative analysis was performed using the chi-square test between the age groups, as seen in Table 3.

Correlation with LI: Among the young, 2.6% of the RE correlated with complex anatomies in the LI, followed by 1.1% in middle-aged individuals. An equal correlation between simple and complex anatomies was seen among the elderly. The chi-square test revealed no statistically significant difference between different age groups in LI (*p* = 0.51), as seen in Table 3.

#### 3.3.3. Site

Table 3 shows that 34 RE (3%) were observed on the right quadrant of the mandible, and 25 (2.2%) were observed on the left.

Correlation with CI: On the right side, 2.1% correlated with CI with complex anatomies, while 1.7% on the left showed a similar correlation. Additionally, 0.9% on the right and 0.5% on the left correlated with CI with simpler anatomy, as seen in Table 3**.**

Correlation with LI: 2% on the right and 1.9% on the left side correlated with LI, with complex anatomies. 1% on the right and 0.4% on the left correlated with LI with simpler anatomies. The chi-square test revealed no statistically significant difference between CI (*p* = 0.64) and LI (*p* = 0.29) concerning the site, as seen in Table 3.

**Figure 4:**
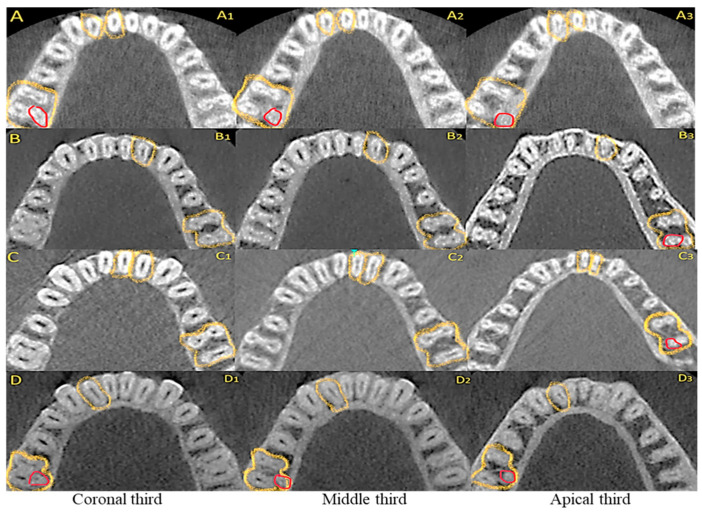
Correlation between distolingual root (DLR) in the mandibular first molar (MFM) and incisor root canal anatomy. (**A**) DLR corresponds with simple anatomy [Vertucci Type I—one canal from the orifice to the apex] in the mandibular incisors represented as (**A1**) in the coronal third, (**A2**) in the middle third and (**A3**) in the apical third; (**B**), DLR in MFM corresponds with complex anatomy [Vertucci Type II—two canals emerging from the orifice (**B1**), merging along its length to end as one canal (**B2**,**B3**) at the apex]; (**C**), DLR in MFM corresponding with complex anatomy in incisors [Vertucci Type III—one canal starting from the orifice (**C1**), dividing into two along its length (**C2**) and then merging into one before exiting the apex (**C3**)]; (**D**), DLR in the MFM corresponds with complex anatomy in incisors [Vertucci Type V—one canal starting from the orifice (**D1**), continuing along its length (**D2**) and dividing into two before exiting the apex (**D3**)]. Note: The DLR is highlighted in red color in the figure. The incisor and the corresponding MFMs with Radix are highlighted in Yellow.

Table 4 presents the relationship between a complex canal system in MIs and the presence of DLR in MFMs on the same side, also representative CBCT images shown in Figure 4.

Complex anatomy was closely linked to DLR, with statistically significant differences observed for both the CI and LI on either side (*p* < 0.01). The odds ratios were statistically significant for either side’s central and lateral incisors. The right central and lateral incisors were 6.92 and 4.33 times more likely to exhibit complex canal anatomy in patients with RE than those without RE. The rather smaller 95% confidence interval revealed better accuracy of results.

Conversely, the odds ratios for MFMs and the left central and lateral incisors (8.56 and 7.93, respectively) point to a significantly stronger probability of encountering complex anatomy. However, the relatively wider confidence interval indicates a lower precision and greater variability among patients exhibiting RE on the left side compared to the right side.

## 4. Discussion

Understanding variations in root canal morphology and the correlations between teeth enhances treatment outcomes [21]. Failure to identify and subsequently clean and seal the root canal space three-dimensionally could result in treatment failure. Missed root canals, due to a lack of knowledge, adequate skill, or anatomical variations, are the largest cause of the onset or persistence of apical periodontitis [1]. In this study, both incisors displayed simple external morphological characteristics (single roots) with canal morphologies varying from simple Vertucci Types I morphology to more complex Vertucci Types III, II, and V. Additional root (DLR) was observed in 5.25% of the MFMs in the samples studied. Furthermore, a strong association was observed between the occurrence of DLR in the MFMs and the complex root canal anatomy in incisors.

Most studies have demonstrated results about the number of roots in mandibular incisors, which aligns with our findings [8,9,10]. Only a couple of studies among Turks and Chinese have previously reported the presence of two roots in MLIs ranging from 0.1 to 0.3% [11,12]. Conversely, variations in their internal anatomy have gained interest from an endodontic point of view, especially the extra canal on the lingual aspect. Failure often occurs because this lingual canal may go unnoticed, as it is typically located beneath the cingulum, which can be challenging to detect with standard radiographs [10]. In this investigation, 71.1% of the MCIs and 64.9% of the MLIs exhibited simple Type I Vertucci canal morphology. More complex canal morphologies were also observed, with Vertucci Type III presenting in 16.2% and 20.8% of MCIs and MLIs, respectively, and Vertucci Type V appearing in 12.7% of MCIs and 10.9% of MLIs. In addition, Type II Vertucci was only observed in 3.4% of MLIs. Similar canal morphologies were noted in previous studies conducted on the Saudi population, with Vertucci Type I reported in approximately 63.5% and 69% of cases in MCIs and MLIs, respectively [22]. Another study in the southern part of the region observed 73.7% Type I morphologies in MCIs and 69.2% Type I morphologies in MLIs [23]. As observed in our study, Vertucci Type III was the most prevalent among the complex canal morphologies identified in these aforementioned investigations. Similarly, in other populations as well, the canal morphologies and percentages of Vertucci Types I and III aligned with the results in our study [8,9]. However, unlike some of the other studies [9], this study did not observe the presence of non-classifiable morphologies, which would have needed a much broader and more incorporating classification system like the one given by Ahmed et al.

The MFMs are typically among the earliest permanent teeth to appear in the mouth. Their uneven occlusal structure leads to a higher risk of decay, which frequently necessitates endodontic treatment. Treating them endodontically is more challenging not only because of their posterior location but also due to their intricate internal characteristics [24]. In most cases, MFMs have two roots, one mesial and one distal. However, variations such as an additional DLR, also known as RE, have been observed in populations worldwide [25]. These variations have been found to have geographic and ethnic origins, with a worldwide prevalence of 5.6–8.9% [25,26]. According to a recent meta-analysis on the global prevalence of DLR, it is often regarded as a typical trait in East Asian populations while uncommon in others, such as Russians, Italians, Venezuelans, and Chileans [26]. The highest reported presence is seen in populations with mongoloid traits like Koreans 27%, Taiwanese 33.3%, and Chinese 31.97% [17,27,28]. In the present study, 5.25% of the MFMs were seen with radix, which aligns with the global average incidence rate. In previous studies, the prevalence of radix in the Saudi population ranged between 3.05 and 6.6%, which aligns with our current study’s findings [29,30,31]. Some variance could be attributed to differences in sample sizes and the assessment methods utilized. Studies conducted in India, Brazil, and Iran have reported incidence rates of radix that align closely with those identified in our research [20,32,33].

Evidence suggests that RE in MFMs is more common in the right quadrant, consistent with our study’s findings, even though this research did not indicate any statistically significant variations [34,35]. Additionally, this investigation did not find differences between genders in the prevalence of RE in MFMs, although it appeared slightly more common in males. These findings are in agreement with other studies that likewise found no gender-related differences [35]. However, Lee et al. and Kim et al. observed a significant prevalence among males in their studies on the Korean population [17,34]. A recent study conducted in Saudi Arabia also found no statistically significant differences in the occurrence of DLR based on site or gender [31]. In terms of age, consistent with past findings in other studies, this research indicated that RE was more common among young people than middle-aged or older adults [19,31]. A significant correlation was observed between RE and canal morphology in MIs, with RE more frequently associated with simpler anatomies in MCIs than complex ones. This finding aligns with Wu et al.’s study in Taiwan [16]. While some researchers attribute similar findings to secondary dentin deposition and calcification in older individuals, our study’s observation of more complex anatomies in younger individuals contradicts this assumption [36]. A more plausible explanation may be the limited representation of older individuals in our study, which could have influenced the discovery of RE in this age group.

Recent investigations have explored potential relationships between the DLR in MFMs and various anatomical features of other teeth. This includes considerations of the C-shaped anatomy found in mandibular second molars, a second mesiobuccal canal (MB2) in maxillary first molars, and the intricate canal systems often seen in mandibular first premolars [35,37,38]. The correlation between root and canal morphologies in MIs and DLR in MFMs was attributed to insights from Papic et al., who suggested that a possible reason for it could be comparable timelines for root completion [39]. Using CBCT, Wu et al. conducted a comprehensive investigation into these correlations within the Taiwanese population, indicating a significant relationship between the complex morphology of MCIs and MLIs and the presence of DLR in MFMs [16,18].

Similar findings and strong associations were also reported by Lee et al. in their study involving the Korean population [17]. Interestingly, the odds ratios for the presence of DLR related to a complex configuration in the incisors were lower than those identified in this study, indicating a significant difference and suggesting an even higher likelihood of correlation among the Saudi population. However, Yang et al., among the Chinese population, identified a positive correlation between the presence of RE and complex anatomy only in MLIs [19]. They noted that this correlation does not extend to complex anatomy in MCIs. In contrast, a study by Pawar et al. found no evidence supporting this correlation among the Indian population [20]. The findings from the aforementioned studies suggest that variations could arise from differences in ethnicity, geographic regions, and sample sizes.

The large number of cases is arguably one of the strengths of the study; nonetheless, there are certain limitations that must be considered when interpreting the findings: 1. Design Limitation: Because of its retrospective design, it is impossible to infer causation between the variables studied. Moreover, as correlations are being tested in this study, causality between variables cannot be proven. Future studies should focus on prospective investigations to determine stronger causal relationships. 2. Single-Center Study: The single-center nature of this study may restrict the generalizability of the results. The samples may not accurately represent the broader population, and the results could be influenced by local factors. Multicenter studies would be essential to enhance the external validity and generalizability of our findings.

## 5. Conclusions

Type I canal morphology was the most common, followed by Type III in both MIs. The interpretations using the Vertucci and Ahmed et al. classification systems were similar; in the current population, no canal morphologies were identified among incisors that could not be classified according to Vertucci’s system. In total, 5.25% of MFMs presented with DLR, which demonstrated a significant association with complex canal morphologies in both MIs. Notably, younger patients exhibited a higher prevalence of such association, while no significant differences were observed based on the site of the MFMs or the gender of the individuals.

## Figures and Tables

**Table 1:** Root canal morphology of Mandibular Incisors—Vertucci and Ahmed et al.

Classification System		Mandibular Incisors (*n* = 2248)				
		Central Incisors *n* = 1124		Lateral Incisors *n* = 1124		Chi-Square Test
Vertucci	Ahmed et al.	41 (%)	31 (%)	42 (%)	32 (%)	
I	^1^TN^1^	402 (71.5)	398 (70.8)	367 (65.4)	362 (64.4)	X^2^ 52.44 *p* <0.001 **
		71.1%		64.9%		
II	^1^TN^2−1^	0	0	22 (3.9)	17 (3)	
				3.4%		
III	^1^TN^1−2−1^	92 (16.4)	90 (16)	116 (20.6)	118 (21)	
		16.2%		20.8%		
V	^1^TN^1−2^	68 (12.1)	74 (13.2)	57 (10.1)	65 (11.6)	
		12.7%		10.9%		

Note: I, Type I Vertucci root canal morphology showing one canal represented as ^1^TN^1^ in Ahmed et al. classification system where superscripted prefix ^1^ represents one root, TN represents the tooth number, suffix ^1^ represents one canal; II, Type II Vertucci root canal morphology showing two canals merging into one represented as ^1^TN^2−1^ in Ahmed et al. classification system where superscripted prefix ^1^ represents one root, TN represents the tooth number, superscripted suffix ^2−1^ represents two canal merging into one; III, Type III Vertucci root canal morphology showing one canal dividing into two and remerging into one, represented as ^1^TN^1−2−1^ in Ahmed et al. classification system where superscripted prefix ^1^ represents one root, TN represents tooth number and suffix ^1−2−1^ represents one canal dividing into two and remerging into one; V, Type V Vertucci root canal morphology showing one canal dividing into two represented as ^1^TN^1−2^ in Ahmed et al. classification system where superscripted suffix ^1^ represents one root, TN represents tooth number and superscripted suffix ^1−2^ represents one canal dividing into two; X^2^ chi-square test; ** highly significant difference statistically with *p* value ≤ 0.05.

**Table 2:** Classifying the mandibular incisors based on the number of canals.

*n*	Canal	Total	Right Central	Left Central	Right Lateral	Left Lateral	Chi-Square
2248 Incisors	Simple	1529 68%	402 71.5%	398 70.8%	367 65.3%	362 64.4%	X^2^ 10.475 *p* 0.014 *
			71.1%		64.9		
	Complex	719 32%	160 28.5%	164 29.2%	195 34.7%	200 35.6%	
			28.85%		35.15%		

Note: *n*, Total number of mandibular incisors; Simple, Vertucci Type I root canal morphology; Complex, Vertucci Types II, III, and V root canal morphologies; X^2^ chi-square test; * statistically significant difference with *p* value ≤ 0.05.

**Table 3:** Distribution and comparative analysis of Radix Entomolaris in mandibular first molars across different demographic groups and incisor anatomies.

Teeth	Type	Gender		Chi-Square Test	Age			Chi-Square Test	Site		Chi-Square Test
		Male	Female		20–39	40–59	>60		Right	Left	
		39	20		38	17	4		34	25	
		3.5%	1.8%		3.4%	1.5%	0.4%		3%	2.2%	
Central incisors	I	9	7	X^2^	7	6	3	X^2^	10	6	X^2^
		0.8%	0.6%	0.95	0.6%	0.5%	0.3%	6.66	0.9%	0.5%	0.21
	III	30	13	*p*	31	11	1	*p*	24	19	*p*
		2.7%	1.2%	0.33 ns	2.8%	1%	0.1%	0.035 *	2.1%	1.7%	0.644 ns
Lateral incisors	I	10	6	X^2^	9	5	2	X^2^	11	5	X^2^
		0.9%	0.5%	0.12	0.8%	0.4%	0.2%	1.33	1%	0.4%	1.112
	III	29	14	*p*	29	12	2	*p*	23	20	*p*
		2.6%	1.3%	0.72 ns	2.6%	1.1%	0.2%	0.51 ns	2%	1.9%	0.29 ns

Note: I, Type I Vertucci root canal morphology showing one canal; III, Type III Vertucci root canal morphology showing one canal dividing into two and remerging into one; X^2^ chi-square test; * statistically significant difference with *p* value ≤ 0.05; ns, non-significant difference.

**Table 4:** The odds ratio for the correlation of DLR with the presence of complex root canal morphology of MIs.

Quadrant	Tooth	Canal	Without DLR	DLR	Total	X^2^	*p*	Odds Ratio	95% CI
Right	Central Incisor	Simple	392	10	402				
			69.75%	1.78%	71.53%	31.52	<0.001 **	6.92	3.22–14.83
		Complex	136	24	160				
			24.19%	4.27%	28.46%				
	Lateral Incisor	Simple	356	11	367	17.34			
			63.34%	1.96%	65.30%		<0.001 **	4.33	2.06–9.08
		Complex	172	23	195				
			30.60%	4.09%	34.69%				
Left	Central Incisor	Simple	392	6	398	27.75			
			69.75%	1.06%	70.81%		<0.001 **	8.56	3.35–21.85
		Complex	145	19	164				
			25.80%	3.38%	29.18%				
	Lateral Incisor	Simple	357	5	362	22.514			
			63.52%	0.88%	64.41%		<0.001 **	7.93	2.92–21.48
		Complex	180	20	200				
			32.08%	3.55%	35.58%				

Note: CI, Confidence interval; DLR, Distolingual root; X^2^, chi-square test; ** highly significant statistical difference with *p* value ≤ 0.05.

## Data Availability

The original contributions presented in this study are included in the article. Further inquiries can be directed to the corresponding author.

## Abbreviations

The following abbreviations are used in this manuscript:

CBCT, Cone Beam Computed Tomography; MFMs, Mandibular First Molars; MIs, Mandibular Incisors; MCIs, Mandibular Central Incisors; MLIs, Mandibular Lateral Incisors; DLR, Distolingual Root; TN, Tooth Number; RCT, Root Canal Treatment; MB2, Second Mesiobuccal Canal.
